# Identification of Oral Secretion Proteins in *Ostrinia furnacalis* by Transcriptome and LC-MS/MS Analyses

**DOI:** 10.3390/insects17040357

**Published:** 2026-03-24

**Authors:** Xinyan Sun, Wei Hu, Dan Wang, Meichen Zhu, Cuiping Xin, Wenbo Yang, Huimin Li, Yanyong Cao

**Affiliations:** State Key Laboratory of Maize Bio-Breeding, Institute of Cereal Crops, Henan Academy of Agricultural Sciences, Zhengzhou 450002, China; xinyansun123@163.com (X.S.); huwei2114@126.com (W.H.); wangdan5567@126.com (D.W.); zmc910824@163.com (M.Z.); xcp18838642579@163.com (C.X.); bbg.123@163.com (W.Y.); lihuimin19910218@126.com (H.L.)

**Keywords:** OS, *O. furnacalis*, effector protein, salivary gland, gut

## Abstract

Oral secretions (OS) of the Asian corn borer (*Ostrinia furnacalis*) play pivotal roles in its interactions with host plants. To systematically characterize the composition and potential functions of OS, 245 functionally annotated proteins from *O. furnacalis* reared on artificial diet were identified using an integrated approach combining transcriptomics and liquid chromatography–tandem mass spectrometry (LC-MS/MS). Bioinformatics analyses were further performed to predict 16 effector proteins. Subsequent tissue-specific expression assays were conducted to quantify the transcriptional levels of genes encoding these effector proteins in salivary glands, guts, and residual tissues. Among these genes, *OfGDH2*, *OfPero2*, *OfPero3*, and *OfCBP* were highly expressed in salivary glands, *OfGDH3*, *OfRGD*, *OfEST1*, *OfEST2*, *OfMET*, and *Offerrin* showed high expression in guts, while *OfSP34* was abundantly expressed in both salivary glands and guts. This study represents the first systematic characterization of the molecular profile of *O. furnacalis* OS, thereby laying a solid foundation for future investigations into the molecular mechanisms underlying the host adaptation of this pest.

## Simple Summary

The Asian corn borer (*Ostrinia furnacalis*), a destructive lepidopteran pest in China’s major maize-producing regions, inflicts severe yield losses via larval boring-feeding across maize key growth stages and induces secondary maize diseases by creating pathogen accessible feeding wounds. As oral secretions (OS) are critical mediators of insect–plant interactions, we identified the component proteins from *O. furnacalis* OS using an integrated transcriptomics–LC-MS/MS approach. Bioinformatics analysis predicted the effector proteins such as protease, dehydrogenase, peroxidase, esterase FE4 that are potentially involved in regulating the pest’s feeding behavior and inducing maize defense responses. This study enriches the understanding of the adaptive mechanisms underlying the interaction between *O. furnacalis* and its plant host, and provides a valuable reference for research on OS of chewing herbivorous insects.

## 1. Introduction

The Asian corn borer (*Ostrinia furnacalis*) is a devastating lepidopteran pest that inflicts severe yield losses on maize production in China through larval boring behavior [1,2,3]. It is widely distributed across all major maize-producing regions of China. Boasting a broad host range, this pest feeds on various economically important crops including maize, sorghum, millet and sugarcane, of which maize incurs the most severe damage. Larvae exhibit a typical boring-feeding habit and infest maize plants throughout key growth stages, from seedling establishment to grain filling. Early instar larvae feed on whorl leaves, whereas late instar larvae bore into stalks, ear pedicels, tassels, and other vital plant organs [4,5]. This feeding behavior not only directly consumes photosynthetic and nutrient-storing tissues, leading to yield losses that typically range from 10% to 30% (and exceed 50% in severely infested fields), but also disrupts the plant’s vascular system via boreholes, inducing stalk breakage and lodging [6,7]. Furthermore, the feeding wounds act as entry points for pathogens causing maize ear rot and stalk base rot, thereby exacerbating the incidence of secondary diseases in infested fields. Coupled with its high fecundity and distinct generation overlapping, this pest is extremely difficult to manage, posing a persistent and grave threat to national food security in China [8,9].

In the long-term coevolution between plants and herbivorous insects, plants have progressively evolved a complex arsenal of anti-herbivore defenses to mitigate damage from feeding, while herbivorous insects have developed diverse counter-defenses to circumvent or overcome plant defenses [10,11,12]. Insect oral secretions (OS), as the primary “molecular messengers” encountered at the insect–host interface, have been widely demonstrated to interact with plant cellular components and participate in defense signaling [13,14,15].

Current research on insect OS focused predominantly on piercing–sucking herbivores, whereas investigations into chewing herbivores remain relatively scarce. The OS of chewing herbivores comprise salivary gland secretions and gut regurgitates, which are derived from distinct tissue sources within the insect body [16]. Saliva is primarily secreted by the mandibular and labial glands, while gut regurgitates originate from the foregut and midgut [17]. These secretions exhibit diverse biological functions, which can be mainly classified into five categories [18]: (1) Detoxification. Herbivorous insects feed on plants containing a variety of toxic secondary metabolites, and their OS harbor numerous detoxification-related enzymes (e.g., cytochrome P450s, dehydrogenases) that mitigate the toxic effects of plant allelochemicals through metabolic transformation [19,20]; (2) Digestion. Digestive enzymes (e.g., amylases, proteases) identified in OS are critical for herbivores to decompose food substrates and assimilate nutrients. For instance, salivary proteases from adult *Heliconius melpomene* facilitate the digestive utilization of pollen nutrients [21]; (3) Regulation of plant defense responses. Insect saliva contains antioxidant enzymes (e.g., peroxidases, catalases) that scavenge reactive oxygen species (ROS) in host plants, thereby reducing the likelihood of herbivore recognition by the host [22,23]; (4) Immunity. The salivary glands of lepidopteran insects secrete immune-related proteins involved in innate immune defense. For example, glucose oxidase (GOX) and lysozyme in the saliva of *Helicoverpa zea* exhibit distinct antibacterial activity, enabling these herbivores to resist invasion by pathogenic microorganisms [24,25]; (5) Modulation of plant volatile emission. OS contain bioactive molecules that regulate the release of host plant volatiles; such modulations can further impair the host plant’s ability to attract natural enemies, thereby indirectly influencing insect survival and reproduction [26].

As a key mediator of plant–insect interactions, insect OS contain both elicitors that can trigger plant defenses and effectors that suppress such response. The latter are crucial for determining the host range of insects and whether they can successfully obtain the required nutrients [27,28]. Thus, the identification of salivary constituents in herbivorous insects is pivotal for screening effector proteins and elucidating the molecular mechanisms underlying plant–insect interactions. In this study, we utilized LC-MS/MS-based proteomics to characterize the constituent profile of larval OS from *O. furnacalis*, and integrated tissue transcriptomic data to annotate the constituents, thereby screening multiple effector proteins that potentially participate in modulating plant defense responses. This work provides direct molecular insights into the interactions between *O. furnacalis* and plants and offers new targets for the development of targeted pest management strategies.

## 2. Materials and Methods

### 2.1. Biological Materials

The *O. furnacalis* populations were purchased from Jiyuan Keyun Biological Co., Ltd. (Jiyuan, China). The insects were reared on artificial diets under a 14 L: 10 D photoperiod at 26 ± 2 °C. To obtain different tissues of insects, we dissected the salivary glands and guts of the 4th instar larvae, and collected these tissues along with residual tissue in 1× PBS buffer. The samples were centrifuged at 12,000 rpm for 5 min, and after removing the supernatant, the remaining samples were stored at −80 °C.

### 2.2. Transcriptome Sequencing

Total RNA was extracted from 50 pairs of salivary glands, 50 residual tissues (salivary glands excluded) from 4th instar larvae using Trizol reagent (15596018, ThermoFisher Scientific, Waltham, MA, USA) according to the manufacturer’s directions. The quantity and purity of total RNA were then assessed using a NanoDrop ND-1000 (ThermoFisher Scientific, Waltham, MA, USA) and RNA integrity was evaluated via Bioanalyzer 2100 (Agilent Technologies, Santa Clara, CA, USA). Use oligo (dT) magnetic beads (25-61005, ThermoFisher Scientific, Waltham, MA, USA) to specifically capture mRNA containing PolyA through two rounds of purification. The captured mRNA was fragmented using the NEBNextR Magnesium RNA Fragmentation Module (New England Biolabs, Ipswich, MA, USA) under high temperature conditions at 94 °C for 5–7 min. cDNA was synthesized from fragmented RNA using reverse transcriptase (1896649, Invitrogen, Carlsbad, CA, USA). Then, cDNA was constructed according to standardized procedures. Finally, we performed double ended sequencing on Illumina Novaseq^TM^ 6000 (Illumina, San Diego, CA, USA) according to standard procedures using PE150 sequencing mode. After obtaining the offline sequencing data, the data was first filtered to obtain high-quality sequencing data (Clean Data) with Cutadapt 1.9 to remove adapters, polyA/polyG regions, reads containing >5% N, and low-quality reads (Q ≤ 20) accounting for >20% of the sequence. Sequence quality was then confirmed using FastQC (v0.11.9, Babraham Institute, Cambs, UK), with key metrics including Q20, Q30, and GC-content of the cleaned data. In addition, these sequences were used as a dedicated reference database for the analysis of raw proteomic data.

### 2.3. Oral Secretions Collection, Protein Extraction and Digestion

For OS collection, the 4th instar larva was gently held between the thumb and forefinger, and the oral cavity was softly touched with a 0.1–10 μL pipette tip [29]. To obtain sufficient protein abundance, three independent sets of OS were collected from larvae across multiple batches and time points. The OS sample was then collected into a tube and stored at −80 °C.

The sample added appropriate amount of 2% sodium deoxycholate lysate and heated at 95 °C for 10 min, broken by ultrasonic breaker (total time 10 min, working for 2 s, stopping for 2 s). And then it was centrifuged 20,000× *g* for 20 min, the supernatant was added 10 mM DTT and bathed in water at 37 °C for 1 h, then 20 mM IAA was added and incubated for 30 min in darkness. Finally, 150 μg protein was taken and 3 μg trypsin added with a ratio of protein: enzyme (50:1), and the samples were incubated for 14–16 h at 37 °C. The enzymatically digested peptides were desalted using waters solid phase extraction cartridges and vacuum dried. The dried peptide fractions were stored at −20 °C.

### 2.4. Nano-LC-MS/MS Analysis

The dried peptide samples were redissolved with 0.1% FA followed by centrifuged at 20,000× *g* for 10 min. The supernatant was collected and injected into a self-loading C18 column. Separation was performed by Thermo Scientific EASY-nLC^TM^ 1200 system (ThermoFisher Scientific, Waltham, MA, USA) at a flow rate of 300 nL/min through the following effective gradient. From 0 to 103 min, 4% solvent B (98% ACN, 0.1% FA) was linearly increased to 27%; 103–111 min, solvent B was increased from 27% to 40%; 111–113 min, solvent B was increased from 40% to 90%; 113–120 min, 90% solvent B. The separated peptides were ionized by a nano-ElectroSpray Ionization and then transferred to Orbitrap Exploris^TM^ 480 mass spectrometer (ThermoFisher Scientific, Waltham, MA, USA) for DDA (Data dependent Acquisition) mode detection. Parameter settings: ion source voltage was 2.2 KV; scan range of primary MS was 350–1500 *m*/*z*; resolution was 60,000; normalized AGC target was 300%, and maximum ion injection time (MIT) was 20 ms; the secondary MS fragmentation mode was HCD. The fragmentation energy was set at 32%; resolution was set at 15,000; dynamic exclusion time was 60 s. The starting *m*/*z* of secondary MS was fixed to 110; the parent ion screening condition for secondary fragmentation was charge 2+ to 6+; normalized AGC target was set at standard, and the MIT was 22 ms.

MaxQuant software (v2.6.6.0, Max Planck Institute of Biochemistry, Martinsried, BY, Germany) was used to analyze the DDA label-free MS/MS data with the following settings: Type: standard; enzyme: Trypsin/P; maximum missed cleavages: 2; fixed modification: carbamidomethyl (C); variable modifications: oxidation (M) and acetyl (protein N-term); precursor mass tolerance: 20 ppm; fragment mass tolerance: 0.05 Da; match between runs and second peptide search was enabled. All other parameters are in default. The MS data were identified with the protein database for transcriptional prediction. The minimum number of unique peptides was set to 1 and FDR threshold was set as 1% at both PSM and protein levels. Protein from contaminant or reverse will be removed.

### 2.5. Bioinformatics Analysis

Differential gene expression analysis was conducted using DESeq2 between two groups. Genes with a false discovery rate (FDR) < 0.05 and absolute fold change ≥ 2 were identified as differentially expressed genes (DEGs). Gene set enrichment analysis (GSEA, v4.1.0) with MSigDB was performed by inputting the gene expression matrix, ranking genes via Signal2Noise normalization, calculating enrichment scores and Q-values with default parameters, and GO terms with |NES| > 1, NOM *p*-value < 0.05 and FDR q-value < 0.25 were considered significantly different between the two groups. Gene Ontology (GO) analysis and Venn analysis were performed using the OmicStudio tools at https://www.omicstudio.cn/tool (accessed on 1 March 2026) [30]. Signal peptide was determined by SignalP 6.0 Server (https://services.healthtech.dtu.dk/services/SignalP-6.0/, accessed on 9 August 2025). SecretomeP-2.0 (https://services.healthtech.dtu.dk/services/SecretomeP-2.0/, accessed on 4 March 2026) was used to predict the non-classical secretion protein. THMHH Server v. 2.0 (https://services.healthtech.dtu.dk/services/TMHMM-2.0/ accessed on 9 August 2025) was used to predict transmembrane helices in proteins.

### 2.6. Quantitative PCR (qPCR) Analysis

The tissues of 20 salivary glands, 20 intestines and 10 residual tissues (salivary glands and intestines excluded) were dissected from 4th instar larvae and three biological replicates were performed. The total RNA was extracted as described above. First-strand cDNA was synthesized using PrimeScript^TM^ RT reagent Kit with gDNA Eraser (RR047A, TAKARA, Kyoto, Japan) to remove genomic DNA. The gene-specific primers were designed using NCBI Primer-BLAST (https://www.ncbi.nlm.nih.gov/tools/primer-blast/index.cgi?LINK_LOC=BlastHome, accessed on 7 January 2026). The *O. furnacalis* housekeeping gene *elongation factor 1-alpha* (*EF1a*, GeneBank accession No. XM_028314033.1) was used as an internal control. The primers are listed in Table S1. Real-time qPCR System using the TB Green^®^ Premix Ex Taq^TM^ II (RR820A, TAKARA, Kyoto, Japan). The reaction program was as follows: an initial denaturation step at 95 °C for 5 min, followed by 40 cycles of 95 °C for 10 s, 60 °C for 20 s and 72 °C 20 s, melt curves stages at 95 °C for 15 s, 60 °C for 1 min, and 95˚C for 15 s. Relative quantification of target genes in different tissues were performed with the 2^−ΔΔCt^ method [31].

## 3. Results

### 3.1. Transcriptome Analysis of O. furnacalis

To achieve a more comprehensive characterization of the OS composition in *O. furnacalis*, cDNA libraries were generated from the salivary glands and residual tissues (salivary glands excluded) using the Illumina Novaseq^TM^ 6000 sequencing platform. Subsequently, transcriptome sequencing was conducted on the six cDNA libraries (three salivary glands replication and three residual tissues replication), which yielded a total of 34.55 G of clean data; among these, the Q30 percentage reached 96.19% (Table S2). Collectively, the transcriptome database established in this study exhibited high quality and reliability, rendering it suitable for subsequent analyses. This database has been deposited in the Sequence Read Archive (SRA) of the National Center for Biotechnology Information (NCBI) with the accession number PRJNA1408563.

**Figure 1:**
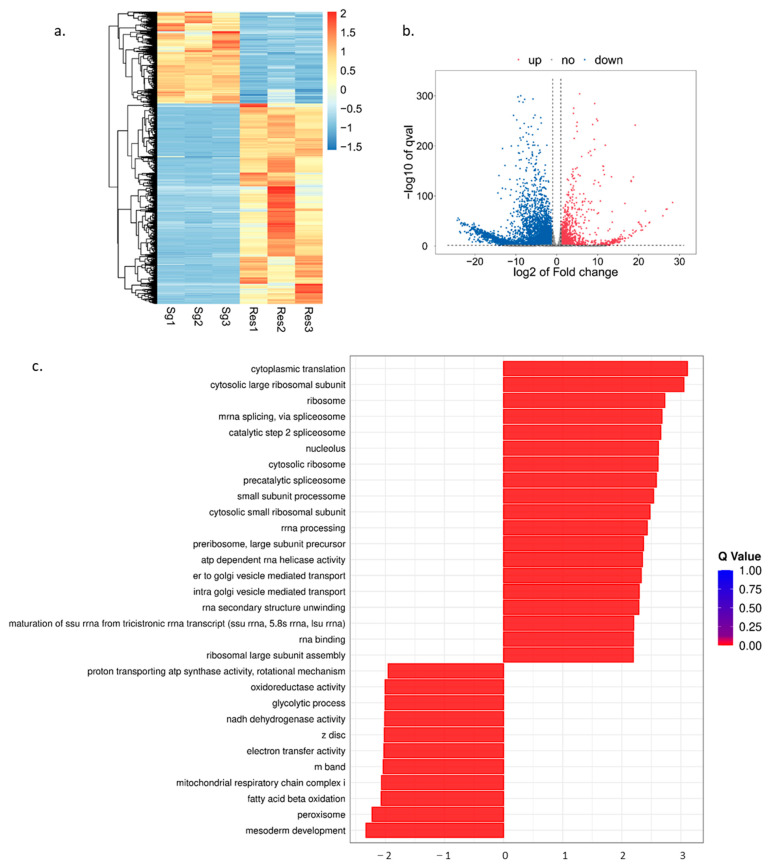
Transcriptome analysis of *O. furnacalis*. (**a**) Heatmap of clustering of gene expression profiles for Sg and Res groups. Sg: salivary gland; Res: residual tissues (salivary gland excluded). (**b**) Volcano plot of differential gene expression between Sg and Res groups. The red dots represent upregulation, the blue dots show downregulation, and the gray dots show no significant changes. (**c**) GSEA enrichment analysis of the DEGs. The bar chart showed the top 30 Q-value terms. Q < 0.05.

A hierarchical clustering heatmap revealed a strong correlation among the samples (Figure 1a). To visualize the distribution of DEGs, a volcano plot was constructed. The results showed that a total of 5812 DEGs were identified in the comparison between the Sg and Res groups, of which 1826 genes were highly expressed in salivary gland tissues and 3986 genes were highly expressed in residual tissues (Figure 1b). Subsequently, GSEA enrichment was performed to characterize the functional features of these genes. The result showed that the top three significant enriched terms in salivary gland tissues included cytoplasmic translation, cytosolic large ribosomal subunit and ribosome, while those in residual tissues were mesoderm development, peroxisome, and fatty acid beta oxidation (Figure 1c).

### 3.2. Proteomic Analysis of OS in O. furnacalis

A total of 316 proteins were identified from *O. furnacalis* OS by LC-MS/MS analysis (Table S3). The LC-MS/MS proteomics data have been publicly deposited in the iProX partner repository with the dataset identifier PXD075177. According to the functions, the identified proteins were divided into six categories: (1) enzymes including oxidoreductases, GTPase, kinase, hydrolases, peptidases, glucosidase, catalase, peroxidase, proteases, transferases, lyases, isomerases, ligases and ATP synthases; (2) transporters including endoplasmic reticulum (ER)-Golgi transporter, Golgi-plasma membrane transport, lipid transporter, protein transporter vesicle transporter, and dsRNA transporter; (3) cytoskeleton protein; (4) DNA-, RNA-, metalion-, chitin-, ATP-, GTP-, carbohydrate- and protein-binding or regulating proteins; (5) other non-enzyme proteins such as ubiquitin, ribosomal protein, organelle protein, serum protein, synapse protein and signal transduction protein; (6) unknown protein.

**Figure 2:**
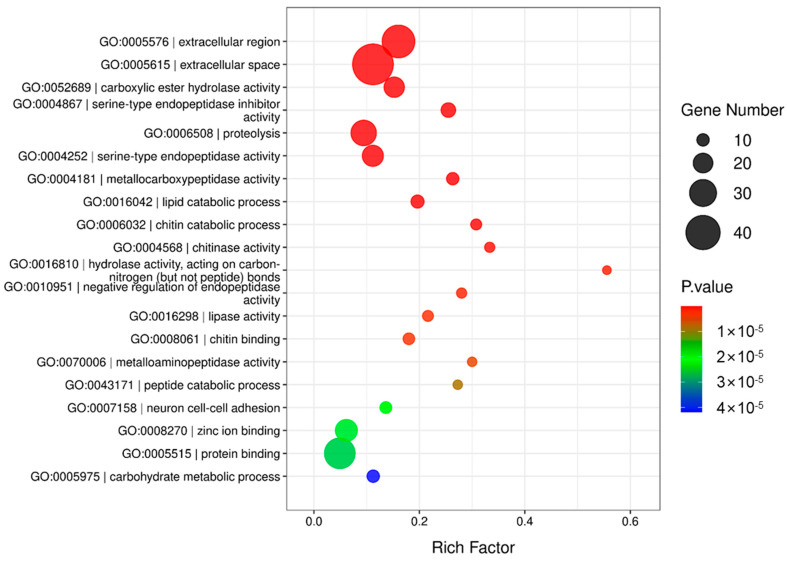
GO enrichment scatter plot analysis of *O. furnacalis* OS proteins.

GO analysis was conducted on the identified proteins. The top 20 representative GO enrichment biological processes are shown in Figure 2. The GO enrichment scatter plot revealed that the most five significantly enriched terms were extracellular region, extracellular space, carboxylic ester hydrolase activity, serine-type endopeptidase inhibitor activity and proteolysis.

### 3.3. Combined Analysis of OS Proteins

**Figure 3:**
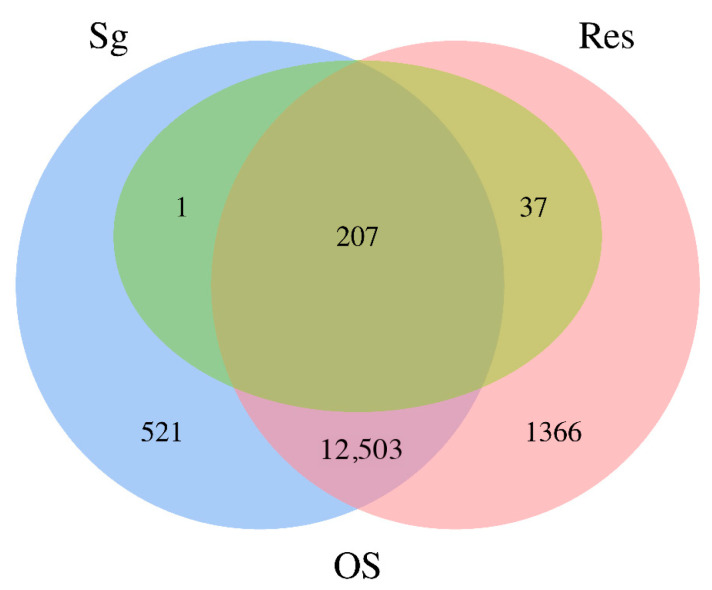
Venn diagram analysis of the relationship among proteins expressed in salivary glands, residual tissues, and oral secretions.

Among the 316 identified proteins, 245 had functional annotations. Subsequent analysis of the transcriptome data generated in our experiments revealed that 207 of these annotated proteins were expressed in both the salivary glands and the residual tissues (saliva proteins and gut regurgitates). Thirty-seven proteins were exclusively expressed in the residual tissues (gut regurgitates), with an FPKM value of 0 in the Sg group, while only 1 protein was uniquely expressed in the salivary glands, with an FPKM value of 0 in the Res group (Figure 3).

Secretory proteins constitute the core component of insect salivary proteins, among which classical secretory proteins secreted via the ER–Golgi pathway are characterized by a single signal peptide and the absence of transmembrane domains [31]. Based on this criterion, we analyzed signal peptides and transmembrane domains of these proteins via bioinformatics. The results showed that 107 proteins expressed both in the salivary glands and residual tissues possessed the structural characteristics of classical secretory proteins (a single signal peptide without transmembrane domains); in contrast, 32 proteins that were exclusively expressed in the residual tissues conformed to these structural features. Since the remaining proteins failed to meet the structural criteria for classical secretory proteins, they were hypothesized to undergo extracellular secretion through non-classical secretory pathways or other unknown mechanisms. Using SecretomeP, we predicted that 34 of these proteins are likely secreted through non-classical secretory pathways (Table S4).

### 3.4. Putative Effector Proteins Prediction

OS released by chewing insects can induce the activation of specific intracellular signaling cascades in host plants. This triggers basal immune responses by modulating cellular signaling such as cytosolic Ca^2+^ elevation, plasma membrane depolarization, ROS and mitogen-activated protein kinase (MAPK) signaling, finally leading to phytohormone-mediated direct and indirect defenses [13,32,33,34,35]. These include various enzymes (metalloproteases, protease inhibitors, clip domain serine proteinases, GOX, lipases, peroxidases and esterases), calcium-binding proteins and odorant-binding chemosensory proteins [27,33]. Based on the functional annotations of the proteins identified in this study, the putative effector proteins we predicted were classified into four categories (enzymes, detoxification proteins, calcium-binding proteins, and other proteins) and listed in Table 1.

### 3.5. Tissue-Specific Expressions of Putative Effector Protein Gene

**Figure 4:**
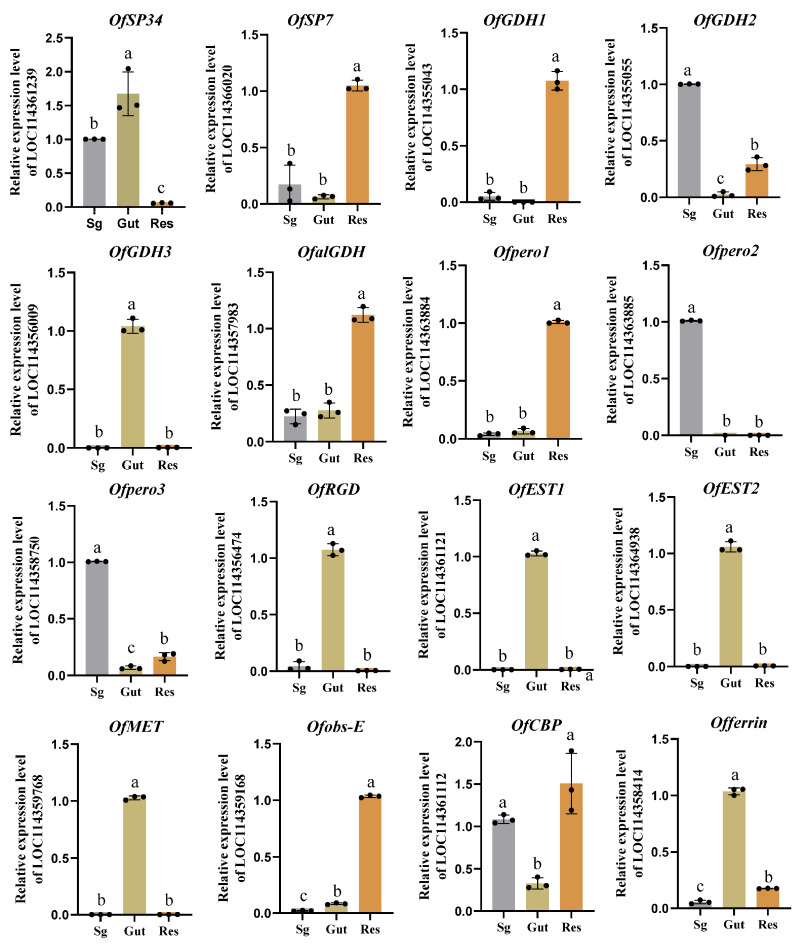
Tissue-specific expression of putative effector protein-encoding genes in *O. furnacalis*. Gene expression was analyzed via qRT-PCR with cDNA from *O. furnacalis* tissues. Means (±SD) represents three replicates. Different letters in the same column in c indicate a significant difference (*p* < 0.05, Tukey’s HSD multiple test). Sg: salivary gland; Gut: gut; Res: residual tissues (salivary gland and gut excluded). The *EF1α* housekeeping gene was used as the internal reference, and relative expression was calculated by the 2^−ΔΔCt^ method.

OS are key factors mediating insect–plant interactions, primarily synthesized and secreted by insect salivary glands and gut tissues [48]. The tissue-specific expression patterns of genes encoding these secreted candidate proteins are closely related to the tissue characteristics and biological functions of their sites of synthesis. Clarifying these expression patterns is a crucial prerequisite for deciphering the functions and mechanisms of such proteins. Therefore, we dissected the salivary glands and guts of *O. furnacalis*, and employed RT-qPCR to detect and analyze the expression levels and distribution characteristics of target genes in the salivary glands, guts, and residual tissues (salivary glands and guts excluded). Notably, *OfGDH2*, *OfPero2*, *OfPero3*, and *OfCBP* were highly expressed in salivary glands, *OfGDH3*, *OfRGD*, *OfEST1*, *OfEST2*, *OfMET*, and *Offerrin* were highly expressed in the guts, while *OfSP34* was abundantly expressed in both the salivary glands and guts (Figure 4).

## 4. Discussion

Herbivorous insect feeding is a pivotal biological event that triggers plant–insect interactions, inducing a systemic response in plants, including a series of physiological and biochemical metabolic changes, cascade activation of cellular signaling pathways, and regulation of defense-related gene expression [27,35]. The OS of herbivorous insects, as key signaling molecules, have been widely confirmed as central factors in activating and precisely regulating plant responses. The current research method for insect OS involves manually collecting complex OS such as insect saliva and gut regurgitant, which are then used as materials for subsequent qualitative and quantitative analysis of components. Combined with in vitro and in vivo experiments, this approach serves as a crucial means to elucidate the molecular mechanisms by which OS mediate plant–insect interactions at the present stage. In this study, we identified 245 functionally annotated proteins using LC-MS/MS. Combined with the transcriptomic results, our analysis revealed that 207 of these proteins were present in both saliva gland and gut regurgitant, 37 proteins originated exclusively from gut regurgitant, and 1 protein derived from salivary gland. Based on the functional annotation information of these proteins, 16 putative effector proteins were initially screened and predicted. With the exception of LOC114363884, which showed high within-group variation in the original expression data and thus no significant difference between the two groups, all other candidate genes exhibited significant differential expression in the transcriptome data (Table S4). Furthermore, our qPCR results showed that *OfSP7*, *OfalGDH*, *Ofpero1*, and *Ofobs-E* exhibited extremely low expression levels in both salivary glands and guts. Given that effector proteins typically require relatively high expression in these tissues to exert their regulatory functions on host plant defenses, proteins with high expression levels in salivary glands or guts are more likely to act as effectors. Therefore, in this study, we further predicted *OfGDH2*, *OfGDH3*, *OfPero2*, *OfPero3*, *OfRGD*, *OfEST1*, *OfEST2*, *OfMET*, *Offerrin*, *OfCBP*, and *OfSP34* as potential effector proteins, which were implicated in insect feeding and plant defense responses.

The OS of insects with chewing mouthparts consist of saliva actively secreted by cephalic glands and gut regurgitant formed by gut contents that are delivered to the oral cavity via retrograde contraction of the esophagus [16,17,49]. Our findings revealed that proteins co-expressed in salivary glands and intestines exhibit extensive functional diversity, encompassing fundamental cellular processes, digestive metabolism, immune defense, and developmental regulation. In contrast, gut-specific proteins display highly specialized functions, primarily dedicated to three core physiological processes: digestive degradation, local immunity, and nutrient utilization. The functional divergence between the two is manifested in multiple aspects. In terms of the digestive enzyme system, co-expressed proteins primarily encode conserved universal digestive enzymes, including carboxypeptidase B-like and trypsin alkaline A/B/C-like, which are capable of degrading common nutrient substrates [50,51]. They also include salivary gland-specific enzymes involved in insect–host interactions (e.g., venom serine protease 34-like, venom dipeptidyl peptidase 4-like) alongside hydrolases (e.g., chitinase, β-N-acetylglucosaminidase) [52,53,54]. Gut-specific proteins, by contrast, encode specialized enzymes (e.g., collagenase, α-amylase) that target complex dietary substrates, thereby functioning as a specialized complement to intestinal digestive processes [55]. Regarding the immune defense system, co-expressed proteins include superoxide dismutase (SOD), catalase, phenoloxidase subunit-like proteins, and serine protease inhibitors. These molecules scavenge oxidative stress products, inhibit a wide range of microorganisms (including bacteria and fungi), and regulate the phenoloxidase cascade, thereby mediating broad-spectrum defense responses [23,56,57,58]. In contrast, gut-specific proteins are enriched exclusively in peptidoglycan recognition proteins (PGRPs). These PGRPs serve as core receptors for the recognition of Gram-positive and Gram-negative bacteria in insects, and specifically modulate the homeostasis of dietary bacteria and intestinal symbiotic flora, preventing dysbiosis and pathogen invasion, thus mediating local targeted immunity [59]. They synergistically modulate key biological processes in insects, including food digestion, host interaction, and survival defense.

Enzymes in insect saliva are key functional molecules mediating interactions between herbivorous insects and their host plants, which hold potential roles in both nutrient digestion and defense regulation, and act as critical candidate factors for insects to adapt to host feeding environments [21,24,27,60,61]. However, direct evidence supporting the regulatory functions of these enzymes is extremely scarce, and their specific modes of action in insect–plant interactions can only be preliminarily inferred through omics-based identification and physiological and biochemical analyses. These enzymes comprise multiple functionally differentiated families. Among them, some proteases may degrade plant cell structural proteins or selectively cleave key signaling components in plant immune pathways, thereby attenuating the host’s defense response; dehydrogenases may scavenge toxic substances generated during the degradation of plant secondary metabolites, thus mitigating the toxicity of plant chemical defenses and maintaining redox homeostasis in insects.

Detoxifying enzymes represent a core functional group for herbivorous insects to overcome plant chemical defenses and achieve feeding adaptation, and relevant research has long been a key focus and hotspot in the field of insect–plant interactions. To date, notable progress has been made in the study of these proteins across multiple detoxifying enzyme families. For carboxylesterases, the research on esterase FE4 has systematically elucidated its multifunctional roles in pesticide metabolism, detoxification of plant ester-based defense and oxidative stress tolerance. Additionally, *esterase FE4* homologous genes in different insect species exhibit species-specific characteristics in host adaptation [41,42,62]. Research on the peroxidase family has focused on the regulation of redox homeostasis and detoxification of phenolic compounds; glutathione peroxidases encoded by *Al6* in *Apolygus lucorum* and *SaE23* in *Sitobion avenae* exert their biological functions by scavenging plant ROS, inhibiting programmed cell death (PCD) and interfering with plant defense signaling pathways [22,39,40]. These studies have clarified the enzymatic characteristics and functional differentiation of core detoxifying enzymes such as carboxylesterases and peroxidases, revealed the molecular basis of their diversification during insect-host coevolution, and also provided a vital research paradigm for deciphering the coordinated regulatory mechanisms of the salivary effector protein network.

Calcium-related proteins in insect saliva are important candidate effector proteins for herbivorous insects to regulate host plant defenses and achieve feeding adaptation [34]. Research on these proteins in piercing–sucking insects, particularly planthoppers, have identified representative members such as EF-hand domain proteins (NlSEF1, LsECP1) and calmodulins. These effectors function through mechanisms including binding host intracellular Ca^2+^, inhibiting ROS bursts and defense hormone pathways, and undermining phloem physical barriers [43,44,63,64]. This potential functional conservation is presumably driven by the conserved requirement of herbivorous insects to counteract host plant Ca^2+^-mediated defense responses—a universal defensive strategy employed by plants against insect herbivory. In contrast, research on salivary calcium-related proteins in chewing insects remains in its early stages and largely relies on homology inference. Based on the functional conservation observed in piercing–sucking insects, it is reasonable to tentatively speculate that calcium-related proteins in the saliva of chewing insects (e.g., regucalcin-like proteins and other calcium-binding proteins in *O. furnacalis*) may share conserved core functions, such as modulating host Ca^2+^ homeostasis and suppressing plant ROS bursts and defense hormone signaling, to facilitate feeding adaptation. Deciphering the effector functions of these proteins provides a crucial theoretical basis for elucidating the damage mechanisms of chewing insects and exploring novel insect-resistant targets against salivary effector proteins.

Although a considerable number of proteins identified in the oral secretions lacked typical N-terminal signal peptides, these proteins could still be released into the extracellular environment through non-classical secretion pathways or other unknown mechanisms. To better characterize their secretory potential, we performed secretion prediction using SecretomeP, which is dedicated to the identification of proteins secreted via non-classical routes [65]. In addition to the classical signal peptide-dependent pathway that traverses the endoplasmic reticulum and Golgi apparatus, non-classical secretion includes mechanisms such as direct transmembrane translocation, export via exosomes or microvesicles, lysosome-mediated release, and passive release upon cell lysis [66,67,68]. These alternative pathways likely account for the presence of proteins without canonical signal peptides in oral secretions, providing a more comprehensive understanding of the secretory landscape in *O. furnacalis*.

This study identified only a small subset of previously reported insect effectors in the saliva of artificially reared *O. furnacalis* larvae. We speculate that this observation stems from the fact that, while artificial diets meet the basic nutritional requirements of larvae, they lack the critical inductive signals that drive effector synthesis and secretion—specifically, those naturally elicited by maize cell wall components, secondary metabolites, and feeding-associated mechanical stimulation. Consequently, the expression abundance of these effectors is significantly reduced. These findings indicate that the artificial diet rearing system has inherent limitations for the identification and functional characterization of salivary effectors, and further validation of the native expression profiles and functional properties of these effectors necessitates integrated analysis with natural host rearing systems. To address this limitation, future studies utilizing maize-fed *O. furnacalis* larvae will complement the present findings through comparative analysis of OS effector profiles between artificially reared and maize-fed larvae, thereby elucidating diet-dependent effector expression patterns. Such comparative analyses enable the discrimination of constitutive OS components that are stably expressed independent of dietary conditions from diet-inducible effectors whose biosynthesis is triggered by interactions with maize tissues or feeding-associated mechanical stimuli. This differentiation enhances our understanding of the regulatory mechanisms governing effector expression and the molecular basis of insect–host interactions; furthermore, data derived from maize-fed larvae will facilitate the validation of the native expression levels and functional significance of these effectors, which in turn helps to overcome the limitations of the artificial diet rearing system.

## Figures and Tables

**Table 1:** Putative effector proteins prediction from proteomic analyses of *O. furnacalis* OS.

Functional Category of Genes	Putative Effector Proteins Proteome	No. of Sequences in Proteome	Gene	References
enzymes	serine protease	2	*LOC114361239*	[27,36,37,38]
			*LOC114366020*	
	metalloprotease	1	*LOC114359768*	
	glucose dehydrogenase	3	*LOC114355043*	
			*LOC114355055*	
			*LOC114356009*	
	aldehyde dehydrogenase	1	*LOC114357983*	
detoxifying proteins	peroxidase like	3	*LOC114363884*	[22,39,40]
			*LOC114363885*	
			*LOC114358750*	
	esterase FE4-like	2	*LOC114361121*	[41,42]
			*LOC114364938*	
calcium-binding proteins	regucalcin-like	1	*LOC114356474*	[43,44]
	calcium-binding protein	1	*LOC114361112*	
other proteins	ferritin	1	*LOC114358414*	[45,46]
	obstructor-E-like	1	*LOC114359168*	[47]

## Data Availability

The data presented in this study are openly available in NCBI at https://www.ncbi.nlm.nih.gov/sra/PRJNA1408563 (accessed on 23 January 2026), reference number PRJNA1408563.

## Supplementary Materials

The following supporting information can be downloaded at: https://www.mdpi.com/article/10.3390/insects17040357/s1, Table S1. The primers of putative effector protein used for qPCR; Table S2. Summary of the *O. furnacalis* transcriptome results; Table S3. Summary of *O. furnacalis* OS proteins identified by LC-MS/MS Analysis; Table S4. Summary of *O. furnacalis* OS proteins identified by combined transcriptome and LC-MS/MS analysis expressed in salivary and gut.
